# {2,2′-[Cyclo­hexane-1,2-diylbis(nitrilo­methanyl­ylidene)]diphenolato}copper(II)

**DOI:** 10.1107/S1600536812036458

**Published:** 2012-08-25

**Authors:** Shunsheng Zhao, Xingqiang Lü, Xiangrong Liu

**Affiliations:** a College of Chemistry and Chemical Engineering, Xi’an University of Science and Technology, Xi’an 710054, Shaanxi, People’s Republic of China; b College of Chemical Engineering, Northwest University, Xi’an 710069, Shaanxi, People’s Republic of China

## Abstract

The title compound, [Cu(C_20_H_20_N_2_O_2_)], crystallizes with two independent mol­ecules in the asymmetric unit. In each mol­ecule, the Cu^II^ atom occupies the tetra­dentate N_2_O_2_ cavity of the salen-type Schiff base ligand, adopting a distorted square-planar geometry with r.m.s. deviations of the coordin­ating atoms of 0.0522 (2) and 0.1128 (4) Å. No hydrogen bonds or π–π stacking inter­action are observed.

## Related literature

For biological activity of salen Schiff bases, see: Caboni *et al.* (2012 ▶). For the synthetic method, see: Marinovich *et al.* (1999 ▶); For related structures, see: Tang (2009 ▶); Ji & Lu (2010 ▶). 
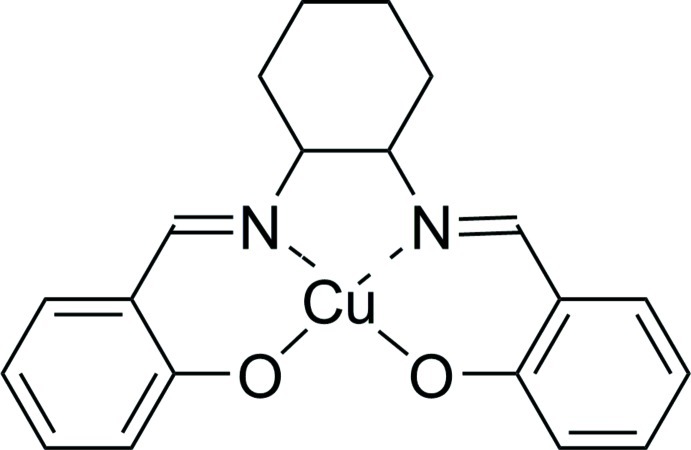


## Experimental

### 

#### Crystal data

[Cu(C_20_H_20_N_2_O_2_)]
*M* *_r_* = 383.93
Monoclinic, 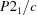

*a* = 12.254 (7) Å
*b* = 24.302 (14) Å
*c* = 12.297 (7) Å
β = 108.654 (9)°
*V* = 3469 (4) Å^3^
*Z* = 8
Mo *K*α radiation
μ = 1.27 mm^−1^
*T* = 296 K
0.38 × 0.24 × 0.21 mm

#### Data collection

Bruker SMART 1K CCD area-detector diffractometer
Absorption correction: multi-scan (*SADABS*; Sheldrick, 2004 ▶) *T*_min_ = 0.700, *T*_max_ = 0.765
16804 measured reflections
6164 independent reflections
2285 reflections with *I* > 2σ(*I*)
*R*_int_ = 0.154

#### Refinement

*R*[*F* ^2^ > 2σ(*F* ^2^)] = 0.085
*wR*(*F* ^2^) = 0.248
*S* = 0.95
6164 reflections
451 parameters
H-atom parameters constrained
Δρ_max_ = 0.59 e Å^−3^
Δρ_min_ = −0.91 e Å^−3^

### 

Data collection: *SMART* (Bruker, 2001 ▶); cell refinement: *SAINT* (Bruker, 2001 ▶); data reduction: *SAINT*; program(s) used to solve structure: *SHELXS97* (Sheldrick, 2008 ▶); program(s) used to refine structure: *SHELXL97* (Sheldrick, 2008 ▶); molecular graphics: *SHELXTL* (Sheldrick, 2008 ▶); software used to prepare material for publication: *SHELXTL*.

## Supplementary Material

Crystal structure: contains datablock(s) I, global. DOI: 10.1107/S1600536812036458/ff2080sup1.cif

Structure factors: contains datablock(s) I. DOI: 10.1107/S1600536812036458/ff2080Isup2.hkl

Additional supplementary materials:  crystallographic information; 3D view; checkCIF report

## Figures and Tables

**Table 1:** Selected bond lengths (Å)

Cu1—O2	1.859 (7)
Cu1—O1	1.897 (6)
Cu1—N1	1.920 (9)
Cu1—N2	1.954 (8)
Cu2—O3	1.868 (6)
Cu2—O4	1.893 (6)
Cu2—N4	1.907 (8)
Cu2—N3	1.929 (7)

**Fig. 1.**
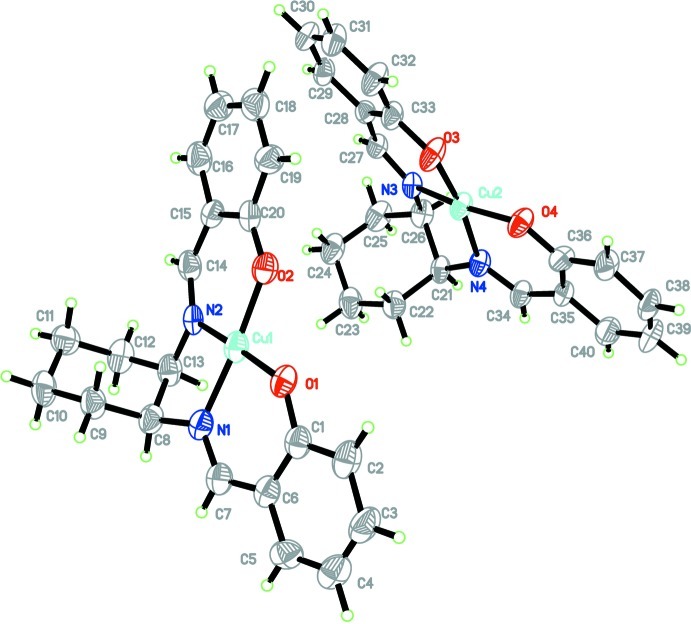
The molecular structure of (I), with atom labels and 50% probability displacement ellipsoids for non-H atoms.

## supplementary crystallographic information

### Comment

Salen Schiff-bases and their metal complexes are of interest due to their biological activity, as well as their optical, catalytic, chromophoric, thermochromic and photochromic properties (Caboni *et al.*, 2012). Here we report the crystal structure of salen-type Schiff-base copper complex. As Fig. 1 shows, the title compound crystallized with two independent molecules in the asymmetric unit. Each of the Cu atoms exists in ann almost planar coordination geometry and is placed in the center of the coordination plane, which is defined by four donor atoms (N1, N2, O1, O2 or N3, N4, O3, O4) of the Schiff-base ligand.

### Experimental

The compound was prepared according to previous reported method (Marinovich *et al.*, 1999). Crystals suitable for the X-ray diffraction study were obtained upon recrystallization from *N,N*-dimethylformamide.

### Refinement

H atoms were positioned geometrically and refined using a riding model with C—H = 0.95–0.99 Å and with *U*_iso_(H) = 1.2 times *U*_eq_(C).

### Crystal data

**Table:**

[Cu(C_20_H_20_N_2_O_2_)]	*F*(000) = 1592
*M**_r_* = 383.93	*D*_x_ = 1.470 Mg m^−^^3^
Monoclinic, *P*2_1_/*c*	Mo *K*α radiation, λ = 0.71073 Å
Hall symbol: -P 2ybc	Cell parameters from 5789 reflections
*a* = 12.254 (7) Å	θ = 1.9–25.3°
*b* = 24.302 (14) Å	µ = 1.27 mm^−^^1^
*c* = 12.297 (7) Å	*T* = 296 K
β = 108.654 (9)°	Block, dark green
*V* = 3469 (4) Å^3^	0.38 × 0.24 × 0.21 mm
*Z* = 8	

### Data collection

**Table:**

Bruker SMART 1K CCD area-detector diffractometer	6164 independent reflections
Radiation source: fine-focus sealed tube	2285 reflections with *I* > 2σ(*I*)
Graphite monochromator	*R*_int_ = 0.154
Thin–slice ω scans	θ_max_ = 25.1°, θ_min_ = 1.7°
Absorption correction: multi-scan (*SADABS*; Sheldrick, 2004)	*h* = −12→14
*T*_min_ = 0.700, *T*_max_ = 0.765	*k* = −28→20
16804 measured reflections	*l* = −14→14

### Refinement

**Table:**

Refinement on *F*^2^	Primary atom site location: structure-invariant direct methods
Least-squares matrix: full	Secondary atom site location: difference Fourier map
*R*[*F*^2^ > 2σ(*F*^2^)] = 0.085	Hydrogen site location: inferred from neighbouring sites
*wR*(*F*^2^) = 0.248	H-atom parameters constrained
*S* = 0.95	*w* = 1/[σ^2^(*F*_o_^2^) + (0.0984*P*)^2^] where *P* = (*F*_o_^2^ + 2*F*_c_^2^)/3
6164 reflections	(Δ/σ)_max_ < 0.001
451 parameters	Δρ_max_ = 0.59 e Å^−^^3^
0 restraints	Δρ_min_ = −0.91 e Å^−^^3^

### Special details

**Table:**

Geometry. All e.s.d.'s (except the e.s.d. in the dihedral angle between two l.s. planes) are estimated using the full covariance matrix. The cell e.s.d.'s are taken into account individually in the estimation of e.s.d.'s in distances, angles and torsion angles; correlations between e.s.d.'s in cell parameters are only used when they are defined by crystal symmetry. An approximate (isotropic) treatment of cell e.s.d.'s is used for estimating e.s.d.'s involving l.s. planes.
Refinement. Refinement of *F*^2^ against ALL reflections. The weighted *R*-factor *wR* and goodness of fit *S* are based on *F*^2^, conventional *R*-factors *R* are based on *F*, with *F* set to zero for negative *F*^2^. The threshold expression of *F*^2^ > σ(*F*^2^) is used only for calculating *R*-factors(gt) *etc*. and is not relevant to the choice of reflections for refinement. *R*-factors based on *F*^2^ are statistically about twice as large as those based on *F*, and *R*- factors based on ALL data will be even larger.

### Fractional atomic coordinates and isotropic or equivalent isotropic displacement parameters (Å^2^)

**Table:**

	*x*	*y*	*z*	*U*_iso_*/*U*_eq_	
Cu2	0.07642 (10)	0.68998 (5)	−0.02055 (9)	0.0691 (5)	
Cu1	0.65609 (10)	0.52703 (5)	−0.00444 (9)	0.0730 (5)	
N3	0.0935 (6)	0.6996 (3)	0.1397 (6)	0.0561 (19)	
O1	0.6408 (6)	0.5123 (3)	−0.1601 (5)	0.0772 (19)	
O4	0.0387 (6)	0.6869 (3)	−0.1822 (5)	0.079 (2)	
O2	0.6494 (5)	0.6020 (3)	−0.0361 (5)	0.0737 (19)	
O3	0.2034 (6)	0.7338 (3)	−0.0150 (5)	0.087 (2)	
N2	0.6614 (6)	0.5371 (4)	0.1548 (6)	0.068 (2)	
N4	−0.0362 (6)	0.6365 (3)	−0.0166 (6)	0.065 (2)	
N1	0.6748 (7)	0.4505 (4)	0.0359 (7)	0.073 (2)	
C27	0.1706 (9)	0.7314 (4)	0.2108 (7)	0.063 (3)	
H9A	0.1679	0.7345	0.2853	0.075*	
C7	0.6501 (8)	0.4090 (5)	−0.0335 (9)	0.075 (3)	
H10A	0.6530	0.3740	−0.0018	0.089*	
C33	0.2746 (10)	0.7626 (4)	0.0725 (7)	0.076 (3)	
C35	−0.1253 (8)	0.6263 (4)	−0.2210 (8)	0.064 (3)	
C28	0.2585 (8)	0.7616 (4)	0.1829 (7)	0.059 (2)	
C6	0.6184 (8)	0.4131 (5)	−0.1561 (9)	0.071 (3)	
C26	0.0031 (9)	0.6724 (4)	0.1748 (7)	0.072 (3)	
H15A	−0.0644	0.6966	0.1523	0.086*	
C29	0.3376 (9)	0.7924 (4)	0.2709 (8)	0.073 (3)	
H16A	0.3287	0.7927	0.3432	0.087*	
C9	0.8436 (9)	0.4491 (4)	0.2040 (8)	0.087 (3)	
H17A	0.8628	0.4844	0.1779	0.104*	
H17B	0.8807	0.4205	0.1735	0.104*	
C36	−0.0496 (10)	0.6608 (4)	−0.2540 (9)	0.074 (3)	
C14	0.6652 (8)	0.5840 (5)	0.2028 (8)	0.077 (3)	
H19A	0.6650	0.5836	0.2783	0.092*	
C30	0.4247 (9)	0.8213 (4)	0.2556 (9)	0.083 (3)	
H20A	0.4754	0.8411	0.3154	0.099*	
C15	0.6698 (8)	0.6367 (5)	0.1546 (8)	0.066 (3)	
C10	0.8874 (10)	0.4472 (5)	0.3359 (9)	0.101 (4)	
H22A	0.9697	0.4541	0.3633	0.121*	
H22B	0.8741	0.4108	0.3616	0.121*	
C34	−0.1134 (8)	0.6147 (4)	−0.1020 (8)	0.071 (3)	
H23A	−0.1647	0.5900	−0.0867	0.085*	
C8	0.7139 (9)	0.4409 (4)	0.1610 (8)	0.079 (3)	
H25A	0.6937	0.4037	0.1786	0.095*	
C20	0.6627 (8)	0.6437 (5)	0.0351 (8)	0.070 (3)	
C21	−0.0296 (8)	0.6198 (4)	0.1019 (7)	0.067 (3)	
H27A	−0.1044	0.6061	0.1029	0.081*	
C25	0.0285 (10)	0.6593 (5)	0.3008 (8)	0.093 (4)	
H28A	−0.0411	0.6460	0.3134	0.112*	
H28B	0.0523	0.6926	0.3456	0.112*	
C32	0.3642 (10)	0.7920 (5)	0.0599 (8)	0.095 (4)	
H29A	0.3757	0.7921	−0.0113	0.114*	
C13	0.6537 (9)	0.4835 (5)	0.2104 (8)	0.080 (3)	
H30A	0.5721	0.4734	0.1866	0.096*	
C22	0.0633 (9)	0.5749 (4)	0.1442 (9)	0.085 (3)	
H31A	0.0370	0.5411	0.1017	0.102*	
H31B	0.1334	0.5864	0.1304	0.102*	
C37	−0.0656 (10)	0.6652 (4)	−0.3730 (8)	0.086 (3)	
H32A	−0.0173	0.6878	−0.3982	0.103*	
C40	−0.2150 (10)	0.5986 (4)	−0.3064 (10)	0.094 (3)	
H33A	−0.2652	0.5757	−0.2845	0.112*	
C38	−0.1520 (10)	0.6365 (5)	−0.4513 (8)	0.089 (3)	
H34A	−0.1582	0.6386	−0.5286	0.106*	
C4	0.5544 (10)	0.3658 (6)	−0.3380 (11)	0.097 (4)	
H35A	0.5330	0.3334	−0.3795	0.117*	
C31	0.4363 (9)	0.8206 (5)	0.1457 (9)	0.089 (3)	
H36A	0.4956	0.8406	0.1323	0.107*	
C5	0.5867 (9)	0.3652 (5)	−0.2215 (10)	0.086 (3)	
H37A	0.5879	0.3318	−0.1841	0.104*	
C11	0.8252 (10)	0.4904 (5)	0.3863 (9)	0.095 (4)	
H38A	0.8523	0.4876	0.4694	0.114*	
H38B	0.8437	0.5269	0.3657	0.114*	
C23	0.0879 (10)	0.5643 (5)	0.2720 (10)	0.100 (4)	
H39A	0.0194	0.5494	0.2844	0.120*	
H39B	0.1486	0.5370	0.2976	0.120*	
C24	0.1237 (11)	0.6157 (5)	0.3420 (9)	0.101 (4)	
H40A	0.1949	0.6296	0.3339	0.121*	
H40B	0.1370	0.6075	0.4224	0.121*	
C2	0.5839 (9)	0.4640 (5)	−0.3347 (8)	0.088 (3)	
H47A	0.5844	0.4965	−0.3745	0.106*	
C19	0.6704 (9)	0.6972 (5)	−0.0004 (10)	0.084 (3)	
H42A	0.6655	0.7028	−0.0767	0.101*	
C1	0.6139 (9)	0.4644 (5)	−0.2133 (9)	0.075 (3)	
C18	0.6845 (9)	0.7424 (5)	0.0686 (11)	0.094 (4)	
H43A	0.6902	0.7774	0.0402	0.113*	
C17	0.6903 (10)	0.7347 (5)	0.1852 (11)	0.097 (4)	
H45A	0.6978	0.7648	0.2337	0.116*	
C16	0.6847 (10)	0.6836 (6)	0.2241 (9)	0.094 (4)	
H44A	0.6909	0.6791	0.3009	0.113*	
C3	0.5537 (10)	0.4146 (6)	−0.3935 (10)	0.102 (4)	
H48A	0.5324	0.4147	−0.4732	0.123*	
C39	−0.2283 (10)	0.6053 (5)	−0.4203 (10)	0.099 (4)	
H49A	−0.2893	0.5885	−0.4759	0.119*	
C12	0.6981 (9)	0.4823 (5)	0.3433 (9)	0.092 (3)	
H50A	0.6609	0.5112	0.3730	0.110*	
H50B	0.6788	0.4473	0.3702	0.110*	

### Atomic displacement parameters (Å^2^)

**Table:**

	*U*^11^	*U*^22^	*U*^33^	*U*^12^	*U*^13^	*U*^23^
Cu2	0.0735 (9)	0.0951 (9)	0.0377 (6)	−0.0100 (8)	0.0163 (5)	−0.0016 (6)
Cu1	0.0678 (9)	0.0969 (10)	0.0496 (7)	0.0040 (8)	0.0120 (6)	0.0010 (6)
N3	0.046 (5)	0.082 (5)	0.044 (4)	0.004 (4)	0.019 (4)	0.006 (4)
O1	0.076 (5)	0.097 (5)	0.051 (4)	−0.007 (4)	0.010 (3)	−0.005 (4)
O4	0.079 (5)	0.108 (5)	0.050 (4)	−0.032 (4)	0.020 (3)	−0.008 (4)
O2	0.077 (5)	0.096 (5)	0.047 (4)	0.001 (4)	0.016 (3)	−0.001 (3)
O3	0.089 (5)	0.145 (6)	0.029 (3)	−0.047 (5)	0.019 (3)	−0.001 (4)
N2	0.052 (5)	0.090 (6)	0.053 (5)	0.012 (5)	0.002 (4)	0.009 (5)
N4	0.060 (5)	0.092 (6)	0.044 (4)	−0.007 (5)	0.019 (4)	0.001 (4)
N1	0.063 (6)	0.093 (6)	0.058 (5)	0.001 (5)	0.012 (4)	−0.006 (5)
C27	0.081 (7)	0.076 (7)	0.029 (4)	0.002 (6)	0.015 (5)	−0.001 (4)
C7	0.052 (6)	0.097 (8)	0.077 (7)	0.001 (6)	0.026 (6)	0.003 (6)
C33	0.083 (8)	0.095 (8)	0.040 (5)	−0.006 (7)	0.007 (5)	0.006 (5)
C35	0.048 (6)	0.081 (7)	0.056 (6)	−0.004 (6)	0.005 (5)	−0.006 (5)
C28	0.062 (6)	0.063 (6)	0.048 (5)	−0.001 (5)	0.012 (5)	0.010 (5)
C6	0.049 (6)	0.101 (8)	0.066 (7)	0.004 (6)	0.023 (5)	−0.011 (7)
C26	0.072 (7)	0.107 (8)	0.046 (5)	0.001 (6)	0.032 (5)	0.001 (5)
C29	0.072 (7)	0.094 (8)	0.046 (5)	0.006 (7)	0.008 (5)	−0.008 (5)
C9	0.088 (9)	0.102 (8)	0.061 (6)	0.018 (7)	0.010 (6)	0.005 (6)
C36	0.077 (8)	0.079 (7)	0.065 (6)	−0.014 (7)	0.022 (6)	−0.007 (6)
C14	0.075 (8)	0.097 (9)	0.053 (6)	0.021 (7)	0.012 (5)	−0.001 (6)
C30	0.060 (7)	0.102 (9)	0.073 (7)	−0.016 (7)	0.005 (6)	−0.013 (6)
C15	0.056 (6)	0.083 (7)	0.054 (6)	0.001 (6)	0.011 (5)	−0.010 (6)
C10	0.094 (9)	0.125 (10)	0.064 (7)	0.010 (8)	0.000 (6)	0.022 (7)
C34	0.055 (6)	0.096 (7)	0.059 (6)	−0.014 (6)	0.015 (5)	0.004 (6)
C8	0.081 (8)	0.091 (8)	0.054 (6)	−0.008 (7)	0.006 (6)	0.008 (6)
C20	0.056 (7)	0.091 (8)	0.056 (6)	0.010 (6)	0.006 (5)	0.006 (6)
C21	0.054 (6)	0.098 (8)	0.048 (5)	−0.002 (6)	0.014 (5)	0.011 (5)
C25	0.097 (9)	0.136 (10)	0.054 (6)	−0.026 (9)	0.035 (6)	0.000 (7)
C32	0.091 (9)	0.140 (10)	0.047 (6)	−0.047 (8)	0.013 (6)	0.009 (6)
C13	0.075 (8)	0.109 (9)	0.053 (6)	0.012 (7)	0.018 (6)	0.003 (6)
C22	0.076 (8)	0.097 (8)	0.075 (7)	−0.006 (7)	0.015 (6)	0.014 (6)
C37	0.107 (9)	0.109 (8)	0.040 (5)	−0.002 (7)	0.021 (6)	−0.002 (6)
C40	0.094 (9)	0.101 (9)	0.076 (8)	−0.013 (7)	0.013 (7)	−0.001 (7)
C38	0.094 (9)	0.117 (9)	0.043 (5)	0.000 (8)	0.004 (6)	−0.017 (6)
C4	0.083 (9)	0.129 (11)	0.086 (9)	0.005 (9)	0.036 (7)	−0.040 (8)
C31	0.072 (7)	0.123 (10)	0.065 (7)	−0.019 (7)	0.012 (6)	0.008 (7)
C5	0.083 (8)	0.087 (8)	0.098 (9)	0.005 (7)	0.041 (7)	−0.016 (7)
C11	0.115 (10)	0.093 (8)	0.071 (7)	0.007 (8)	0.022 (7)	0.005 (6)
C23	0.084 (8)	0.122 (10)	0.085 (8)	−0.008 (8)	0.016 (7)	0.039 (8)
C24	0.102 (10)	0.132 (10)	0.059 (7)	−0.030 (9)	0.012 (7)	0.021 (7)
C2	0.081 (8)	0.133 (10)	0.051 (6)	0.000 (8)	0.023 (6)	−0.009 (7)
C19	0.092 (9)	0.083 (8)	0.075 (7)	−0.007 (7)	0.022 (6)	−0.003 (7)
C1	0.059 (7)	0.101 (9)	0.066 (7)	0.006 (7)	0.020 (6)	−0.003 (7)
C18	0.075 (8)	0.102 (10)	0.100 (10)	−0.005 (7)	0.019 (7)	0.014 (8)
C17	0.092 (9)	0.095 (9)	0.095 (10)	0.004 (8)	0.016 (7)	−0.018 (8)
C16	0.096 (9)	0.116 (10)	0.061 (7)	0.020 (9)	0.010 (6)	−0.002 (8)
C3	0.094 (9)	0.136 (11)	0.068 (8)	0.002 (9)	0.015 (7)	−0.040 (9)
C39	0.079 (9)	0.139 (11)	0.061 (7)	−0.019 (8)	−0.002 (6)	−0.023 (7)
C12	0.074 (8)	0.132 (10)	0.071 (7)	−0.004 (7)	0.025 (6)	0.007 (7)

### Geometric parameters (Å, º)

**Table:**

Cu2—O3	1.868 (6)	C10—H22B	0.9700
Cu2—O4	1.893 (6)	C34—H23A	0.9300
Cu2—N4	1.907 (8)	C8—C13	1.507 (13)
Cu2—N3	1.929 (7)	C8—H25A	0.9800
Cu1—O2	1.859 (7)	C20—C19	1.386 (13)
Cu1—O1	1.897 (6)	C21—C22	1.543 (12)
Cu1—N1	1.920 (9)	C21—H27A	0.9800
Cu1—N2	1.954 (8)	C25—C24	1.537 (14)
N3—C27	1.312 (10)	C25—H28A	0.9700
N3—C26	1.468 (11)	C25—H28B	0.9700
O1—C1	1.325 (11)	C32—C31	1.336 (13)
O4—C36	1.320 (11)	C32—H29A	0.9300
O2—C20	1.315 (11)	C13—C12	1.548 (12)
O3—C33	1.345 (11)	C13—H30A	0.9800
N2—C14	1.278 (11)	C22—C23	1.524 (13)
N2—C13	1.488 (12)	C22—H31A	0.9700
N4—C34	1.283 (10)	C22—H31B	0.9700
N4—C21	1.489 (10)	C37—C38	1.372 (13)
N1—C7	1.293 (11)	C37—H32A	0.9300
N1—C8	1.475 (11)	C40—C39	1.367 (14)
C27—C28	1.434 (12)	C40—H33A	0.9300
C27—H9A	0.9300	C38—C39	1.350 (14)
C7—C6	1.435 (12)	C38—H34A	0.9300
C7—H10A	0.9300	C4—C5	1.359 (14)
C33—C32	1.359 (13)	C4—C3	1.366 (15)
C33—C28	1.434 (12)	C4—H35A	0.9300
C35—C36	1.404 (12)	C31—H36A	0.9300
C35—C40	1.423 (13)	C5—H37A	0.9300
C35—C34	1.451 (12)	C11—C12	1.489 (13)
C28—C29	1.414 (12)	C11—H38A	0.9700
C6—C5	1.398 (13)	C11—H38B	0.9700
C6—C1	1.423 (13)	C23—C24	1.501 (14)
C26—C25	1.514 (11)	C23—H39A	0.9700
C26—C21	1.539 (12)	C23—H39B	0.9700
C26—H15A	0.9800	C24—H40A	0.9700
C29—C30	1.340 (13)	C24—H40B	0.9700
C29—H16A	0.9300	C2—C3	1.390 (14)
C9—C8	1.519 (13)	C2—C1	1.418 (13)
C9—C10	1.537 (12)	C2—H47A	0.9300
C9—H17A	0.9700	C19—C18	1.364 (14)
C9—H17B	0.9700	C19—H42A	0.9300
C36—C37	1.417 (12)	C18—C17	1.425 (15)
C14—C15	1.418 (13)	C18—H43A	0.9300
C14—H19A	0.9300	C17—C16	1.340 (14)
C30—C31	1.404 (13)	C17—H45A	0.9300
C30—H20A	0.9300	C16—H44A	0.9300
C15—C16	1.402 (13)	C3—H48A	0.9300
C15—C20	1.454 (12)	C39—H49A	0.9300
C10—C11	1.539 (14)	C12—H50A	0.9700
C10—H22A	0.9700	C12—H50B	0.9700
			
O3—Cu2—O4	89.0 (3)	N4—C21—C22	109.0 (7)
O3—Cu2—N4	171.1 (3)	C26—C21—C22	111.5 (8)
O4—Cu2—N4	93.7 (3)	N4—C21—H27A	110.3
O3—Cu2—N3	94.4 (3)	C26—C21—H27A	110.3
O4—Cu2—N3	171.2 (3)	C22—C21—H27A	110.3
N4—Cu2—N3	84.2 (3)	C26—C25—C24	111.6 (9)
O2—Cu1—O1	89.4 (3)	C26—C25—H28A	109.3
O2—Cu1—N1	175.6 (3)	C24—C25—H28A	109.3
O1—Cu1—N1	92.5 (3)	C26—C25—H28B	109.3
O2—Cu1—N2	94.1 (3)	C24—C25—H28B	109.3
O1—Cu1—N2	174.9 (3)	H28A—C25—H28B	108.0
N1—Cu1—N2	84.2 (4)	C31—C32—C33	122.6 (11)
C27—N3—C26	121.2 (7)	C31—C32—H29A	118.7
C27—N3—Cu2	124.9 (6)	C33—C32—H29A	118.7
C26—N3—Cu2	113.6 (5)	N2—C13—C8	107.8 (8)
C1—O1—Cu1	126.3 (6)	N2—C13—C12	116.7 (9)
C36—O4—Cu2	126.4 (6)	C8—C13—C12	111.1 (8)
C20—O2—Cu1	128.8 (6)	N2—C13—H30A	106.9
C33—O3—Cu2	130.3 (6)	C8—C13—H30A	106.9
C14—N2—C13	124.6 (9)	C12—C13—H30A	106.9
C14—N2—Cu1	124.0 (7)	C23—C22—C21	110.4 (9)
C13—N2—Cu1	111.3 (6)	C23—C22—H31A	109.6
C34—N4—C21	118.9 (8)	C21—C22—H31A	109.6
C34—N4—Cu2	127.7 (7)	C23—C22—H31B	109.6
C21—N4—Cu2	113.4 (6)	C21—C22—H31B	109.6
C7—N1—C8	119.7 (9)	H31A—C22—H31B	108.1
C7—N1—Cu1	127.0 (7)	C38—C37—C36	120.5 (11)
C8—N1—Cu1	113.2 (7)	C38—C37—H32A	119.8
N3—C27—C28	125.3 (8)	C36—C37—H32A	119.8
N3—C27—H9A	117.4	C39—C40—C35	120.7 (11)
C28—C27—H9A	117.4	C39—C40—H33A	119.6
N1—C7—C6	124.5 (10)	C35—C40—H33A	119.6
N1—C7—H10A	117.8	C39—C38—C37	122.4 (10)
C6—C7—H10A	117.8	C39—C38—H34A	118.8
O3—C33—C32	121.6 (9)	C37—C38—H34A	118.8
O3—C33—C28	119.8 (10)	C5—C4—C3	119.3 (12)
C32—C33—C28	118.6 (9)	C5—C4—H35A	120.3
C36—C35—C40	119.7 (10)	C3—C4—H35A	120.3
C36—C35—C34	123.0 (8)	C32—C31—C30	121.4 (11)
C40—C35—C34	117.2 (10)	C32—C31—H36A	119.3
C29—C28—C27	118.2 (9)	C30—C31—H36A	119.3
C29—C28—C33	116.5 (9)	C4—C5—C6	122.1 (11)
C27—C28—C33	125.3 (9)	C4—C5—H37A	119.0
C5—C6—C1	119.0 (10)	C6—C5—H37A	119.0
C5—C6—C7	118.6 (11)	C12—C11—C10	111.3 (10)
C1—C6—C7	122.4 (10)	C12—C11—H38A	109.4
N3—C26—C25	117.9 (8)	C10—C11—H38A	109.4
N3—C26—C21	106.4 (7)	C12—C11—H38B	109.4
C25—C26—C21	110.6 (8)	C10—C11—H38B	109.4
N3—C26—H15A	107.1	H38A—C11—H38B	108.0
C25—C26—H15A	107.1	C24—C23—C22	112.1 (9)
C21—C26—H15A	107.1	C24—C23—H39A	109.2
C30—C29—C28	123.5 (10)	C22—C23—H39A	109.2
C30—C29—H16A	118.2	C24—C23—H39B	109.2
C28—C29—H16A	118.2	C22—C23—H39B	109.2
C8—C9—C10	109.7 (9)	H39A—C23—H39B	107.9
C8—C9—H17A	109.7	C23—C24—C25	109.5 (9)
C10—C9—H17A	109.7	C23—C24—H40A	109.8
C8—C9—H17B	109.7	C25—C24—H40A	109.8
C10—C9—H17B	109.7	C23—C24—H40B	109.8
H17A—C9—H17B	108.2	C25—C24—H40B	109.8
O4—C36—C35	124.8 (9)	H40A—C24—H40B	108.2
O4—C36—C37	117.9 (10)	C3—C2—C1	119.3 (11)
C35—C36—C37	117.3 (9)	C3—C2—H47A	120.3
N2—C14—C15	127.8 (9)	C1—C2—H47A	120.3
N2—C14—H19A	116.1	C18—C19—C20	124.6 (11)
C15—C14—H19A	116.1	C18—C19—H42A	117.7
C29—C30—C31	117.3 (10)	C20—C19—H42A	117.7
C29—C30—H20A	121.3	O1—C1—C2	117.7 (10)
C31—C30—H20A	121.3	O1—C1—C6	124.1 (9)
C16—C15—C14	119.6 (10)	C2—C1—C6	118.1 (10)
C16—C15—C20	118.3 (10)	C19—C18—C17	118.5 (11)
C14—C15—C20	122.0 (9)	C19—C18—H43A	120.8
C9—C10—C11	110.7 (8)	C17—C18—H43A	120.8
C9—C10—H22A	109.5	C16—C17—C18	119.2 (11)
C11—C10—H22A	109.5	C16—C17—H45A	120.4
C9—C10—H22B	109.5	C18—C17—H45A	120.4
C11—C10—H22B	109.5	C17—C16—C15	123.2 (11)
H22A—C10—H22B	108.1	C17—C16—H44A	118.4
N4—C34—C35	123.7 (9)	C15—C16—H44A	118.4
N4—C34—H23A	118.2	C4—C3—C2	122.1 (11)
C35—C34—H23A	118.2	C4—C3—H48A	119.0
N1—C8—C13	105.9 (8)	C2—C3—H48A	119.0
N1—C8—C9	107.3 (8)	C38—C39—C40	119.3 (10)
C13—C8—C9	111.2 (9)	C38—C39—H49A	120.3
N1—C8—H25A	110.7	C40—C39—H49A	120.3
C13—C8—H25A	110.7	C11—C12—C13	110.3 (9)
C9—C8—H25A	110.7	C11—C12—H50A	109.6
O2—C20—C19	121.3 (9)	C13—C12—H50A	109.6
O2—C20—C15	122.5 (10)	C11—C12—H50B	109.6
C19—C20—C15	116.1 (10)	C13—C12—H50B	109.6
N4—C21—C26	105.5 (7)	H50A—C12—H50B	108.1
